# An Automated Microfluidic System for the Generation of Droplet Interface Bilayer Networks

**DOI:** 10.3390/mi8030093

**Published:** 2017-03-21

**Authors:** Magdalena A. Czekalska, Tomasz S. Kaminski, Michal Horka, Slawomir Jakiela, Piotr Garstecki

**Affiliations:** 1 Institute of Physical Chemistry, Polish Academy of Sciences, Kasprzaka 44/52, 01-224 Warsaw, Poland; mczekalska@ichf.edu.pl (M.A.C.); tkaminski@ichf.edu.pl (T.S.K.); mhorka@ichf.edu.pl (M.H.); 2 Department of Biophysics, Warsaw University of Life Sciences, Nowoursynowska 159, 02-776 Warsaw, Poland; slawomir_jakiela@sggw.pl

**Keywords:** microfluidics, microdroplets, droplet interface bilayers (DIBs), droplet networks, electrophysiology, model lipid membrane

## Abstract

Networks of droplets, in which aqueous compartments are separated by lipid bilayers, have shown great potential as a model for biological transmembrane communication. We present a microfluidic system which allows for on-demand generation of droplets that are hydrodynamically locked in a trapping structure. As a result, the system enables the formation of a network of four droplets connected via lipid bilayers and the positions of each droplet in the network can be controlled thanks to automation of microfluidic operations. We perform electrophysiological measurements of ionic currents indicating interactions between nanopores and small molecules to prove the potential of the device in screening of the inhibitors acting on membrane proteins. We also demonstrate, for the first time, a microfluidic droplet interface bilayer (DIB) system in which the testing of inhibitors can be performed without direct contact between the tested sample and the electrodes recording picoampere currents.

## 1. Introduction

Stable and functional phospholipid bilayers can be easily formed using the Droplet Interface Bilayer (DIB) method—a simple experimental approach that recently gained attention due to its broad capabilities in the field of synthetic biology [1,2]. The DIB technique relies on the self-organization of phospholipid molecules at the interface of the aqueous droplets and continuous phase (usually organic oils are used) and subsequent formation of the bilayer when monolayers of two droplets are brought into intimate contact. Networks of aqueous droplets separated by lipid bilayers have shown great potential as a model for biological transmembrane communication. In particular, networks of three or more droplets that can chemically communicate with each other by membrane proteins, may show collective behavior and exhibit properties such as light sensing or the production of energy in synthetic “biobatteries” [3,4,5,6,7,8].

Droplet microfluidics is a technology that offers several advantages in a rapid and automated assembly of droplet networks. Recent inventions of dedicated structures and geometries on a chip, such as rails [9] or traps [10,11,12,13,14] allowed for an ordered assembly of nanoliter droplets into networks, in which a chemical signal was propagating via the transport of molecules through lipid bilayers. An interesting approach was recently demonstrated by Villar et al. [15] who printed out a 3D network composed of tens of thousands of picoliter droplets—a structure that resembles a tissue-like material.

So far, in comparison to the manual handling of nano- and microliter droplets, microfluidic techniques for the preparation of droplet networks suffer from few limitations [16]. Individual droplets could not be addressed (e.g., removed from the network). Furthermore, most microfluidic studies relied on the fluorescent monitoring of the diffusion of small molecules between the compartments [9,17,18], rather than electrophysiological measurements of ionic currents [13,14,19,20]. Importantly, the electrophysiological measurements require direct contact of an electrode with the measured sample containing tested proteins or inhibitors. So far, this problem has been solved by cyclic washing of electrodes with pure buffer [14] or by fabrication of arrays comprising multiple electrodes [21,22,23].

**Figure 1:**
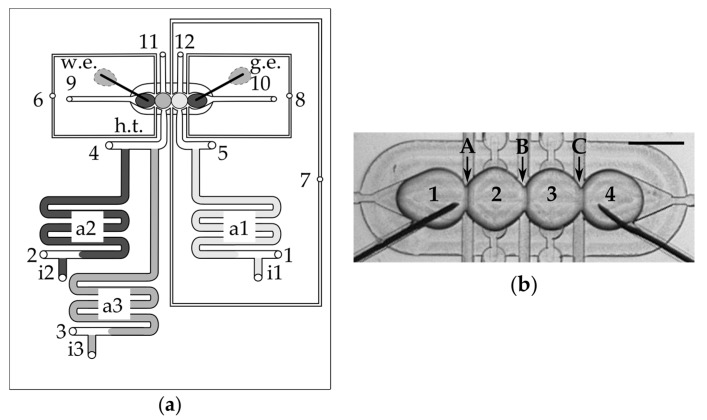
(**a**) Scheme of the microfluidic chip: “1–8”—inlets for oil; “9–12”—outlets; “a1–a3”—aspiration modules for aqueous samples; “i”—inlets for aqueous samples; “i1”—buffer/solution of inhibitor; “i2”—solution of α-hemolysin (300 nM); “i3”—solution of α-hemolysin (3 nM); “w.e.”—working electrode; “g.e.”—ground electrode; “h.t.”—hydrodynamic trap. All inlets and outlets are interfaced with tubing and capillaries via short steel needles, inserted into the circular through holes, milled in the layers of the chip. (**b**) A micrograph of the hydrodynamic trap comprising a network of four droplets (“1–4”, 1 M KCl, 10 mM Hepes, pH 7) interconnected with DPhPC bilayers (marked as A–C). The scale bar is 1 mm.

Here, we address the aforementioned limitations by designing and fabricating a microfluidic system, enabling the generation of networks of aqueous droplets in a controlled way. Aqueous droplets are first generated on demand [24] and then assembled into a linear network locked in the hydrodynamic trap. The droplets are submerged in oil comprising dissolved phospholipids. We actively control the flow of liquids on a chip, in order to transport droplets into a special hydrodynamic structure of channels, a so-called “trap” [25]. In the trap, four droplets are arranged in a line to form a network comprising three bilayers at the interfaces: (i) outer bilayer A between the first and second droplet; (ii) middle bilayer B between the second and third droplet and (iii) outer bilayer C between the third and fourth droplet (Figure 1). We are able to regulate the contact surface between the droplets and remove one or both of the inner droplets from the network on demand.

We are also able to study the electrical properties of the DIBs network via electrodes integrated on the chip. The silver-chloride wires penetrate the outer compartments of the networks—droplets containing a high concentration of α-hemolysin nanopores. A large number of protein nanopores spontaneously insert into bilayers separating the outer and inner droplets. As a result, high perforation of the lipid membrane reduces the electric resistance of the outer bilayers and allows for insight into the electrical processes that take part at the interface of the inner droplets [26]—the middle bilayer of the network. The physical separation of the outer droplets that host the electrodes from the inner (test) droplets allows us to perform a rapid screening of the interactions of small molecules with the single protein molecule, without needing to wash the electrodes between exchanges of the samples.

## 2. Materials and Methods

### 2.1. Reagents

1,2-Diphytanoyl-*sn*-glycero-3-phosphocholine (DPhPC, Avanti Polar Lipids, Alabaster, AL, USA), hexadecane (Sigma Aldrich, Darmstadt, Germany), silicone oil AR20 (Sigma Aldrich), α-hemolysin (Sigma Aldrich) and γ-cyclodextrin (Cyclolab, Budapest, Hungary) were used as received. The buffer for protein and γ-cyclodextrin dilution consisted of 1 M KCl (Sigma Aldrich) and 10 mM Tris–HCl (Roth, Karlsruhe, Germany), pH 7.0. The lipid solution was prepared by dissolving DPhPC (200 mg) in chloroform (10 mL, Chempur, Piekary Slaskie, Poland). The chloroform was evaporated under vacuum and the lipid film was re-solubilized in a mixture of 75% (*v*/*v*) hexadecane (Alfa Aesar, Karlsruhe, Germany) and 25% (*v*/*v*) silicone oil AR20 (Sigma Aldrich) to the final concentration 1 mg/mL.

### 2.2. Microchip Fabrication

We fabricated the polycarbonate chip from 5-mm-thick plates (Makrolon, Bayer, Leverkusen, Germany) using a CNC milling machine (MSG4025, Ergwind, Gdansk, Poland). The two milled plates were thermally bonded by compressing them together for 30 min at 130 °C. We inserted 15 steel needles (4-cm-long, outer diameter (O.D.) 0.82 mm, internal diameter (I.D.) 0.65 mm, Fishman Corporation, Hopkinton, MA, USA) into the 0.8 mm through holes which served as inlets. We used the resistive steel capillaries (O.D. 0.4 mm, I.D. 0.205 mm, length 100 cm) to connect the device to the external electromagnetic valves (Sirai, Bussero, Italy). Eight needles (inlets Nos. 1–8 in Figure 1a) were connected to capillaries using segments of Tygon tubing (~2 cm, O.D. 0.91 mm, I.D. 0.25 mm, Ismatec, Wertheim, Germany). Three other needles (inlets Nos. i1–i3) were connected through PTFE tubing (O.D. 1.6 mm, I.D. 0.8 mm, Bola Bohlender, Grünsfeld, Germany) with 500 μL syringes, each equipped with a built-in valve (1750SL Gastight, Hamilton, Reno, NE, USA), which were used to store and introduce aqueous solution. Finally, the last four needles (Nos. 9–12 in Figure 1), which served as outlets, were connected with valves via 50 cm PTFE tubing (O.D. 1.6 mm, I.D. 0.8 mm, Bola Bohlender).

### 2.3. Electrical Recordings

Ag/AgCl electrodes were used for electrical measurements. Silver wires, 100 μm in diameter, (Sigma Aldrich) were treated overnight (12 h) with sodium hypochlorite solution (Sigma Aldrich). The tips of the electrodes were covered with agarose (1% *w*/*v*, Roth) containing 1 M KCl and 10 mM Tris–HCl. An Axopatch 200B patch-clamp amplifier (Molecular Devices, Sunnyvale, CA, USA) was used for recording the electrical current, which was acquired with a 1 kHz low-pass Bessel filter at a sampling rate of 10 kHz.

## 3. Results and Discussion

### 3.1. Layout and Operation of the Device

The width and depth of the milled microfluidic channels is 400 μm (excluding channels originated from inlets Nos. 6–8 which have a width of 200 μm—see Figure 1a and Figure S1). The device comprises a microfluidic trap in which droplets are brought into contact to form DIBs. The structure of the trap comprises four cavities surrounded by a shallower, oval area—the so-called “bypass”. The purpose of the bypass is to allow for small flows of oil around the droplets without distorting their position. There are three sample ports with T-junctions for storage of samples on a chip. Silver wire electrodes were introduced into the trap prior to running an assay.

The chip was operated in a similar way to the simpler, 8-valve system [14] that allowed for the creation of only a single DIB between a pair of droplets. Briefly, the chip was first filled with oil and then aqueous solutions of buffer and samples with dissolved protein or inhibitor were introduced into the storage channels (see Figure S1 for more details). The droplets with volume of app. 500 nL were first generated at the T-junctions and next they were transported to the trap in which droplets are hydrodynamically locked by the capillary force.

### 3.2. Droplets Form a Network in a Trap

**Figure 2:**
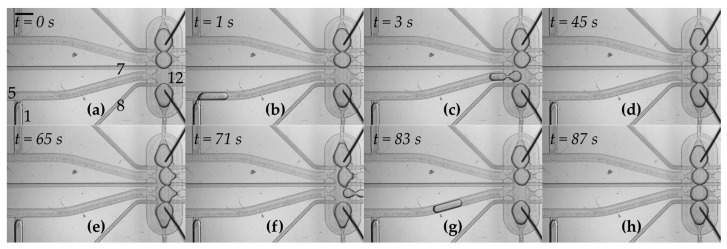
Formation of the network of droplets and exchange of one of the components—snapshots from Video S1. The scale bar is 1 mm. (**a**) Initial state in which three droplets are already positioned within the hydrodynamic trap. The numbers represent respective valves which are used in the following steps. Outlet No. 12 remains open during operations; (**b**) a droplet is generated at the T-junction. The aqueous plug is pushed by applying the flow from valve No. 1; (**c**) oil applied from valve No. 5 pushes the droplet into the trap; (**d**) after about 60 s of incubation, droplet interface bilayers are formed within the network; (**e**,**f**) the flow of oil from the thin perpendicular channels (applied from valves No. 7 and No. 8 at the same time) pushes the droplet from the trap into outlet No. 12; (**g**,**h**) a new droplet is generated and transferred into the trap. It takes approximately 4 s to generate and transfer a new droplet into the trap, and about 7 s to remove one of the droplets from the network.

Nanoliter droplets were sequentially transported to the microfluidic trap, positioned next to each other thanks to the structure of the trap and after about 60 s the droplet interface bilayers from DPhPC (1 mg/mL) were spontaneously formed (Figure 2, Video S1). The flow rate was approximately 280 nL·s^−1^. The operations such as formation of droplets, their transportation and removal on demand took less than 20 s all together.

**Figure 3:**
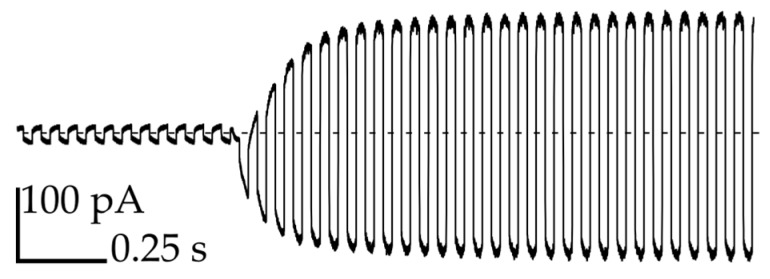
Measurement of the capacitive current during the formation process of the bilayer between droplet No. 3 and the neighboring droplets.

In order to confirm the formation of bilayers, we applied a triangular potential (10 Hz, 50 mV peak to peak) and recorded the resulting square wave capacitive current at 1 kHz sampling rate (Figure 3). A rapid increase in the current is attributed to the formation of bilayers that exhibit high electric capacity [27]. We applied the flow of oil from thin channels (perpendicular to the axis of the network) in order to regulate the size of the bilayer or separate the droplets on demand in a controlled manner (see Video S2 and Figure S2) [14]. The narrow cross-section of the perpendicular channels ensures an independent application of the stream of oil in the area exactly between the chosen pair of droplets.

**Figure 4:**
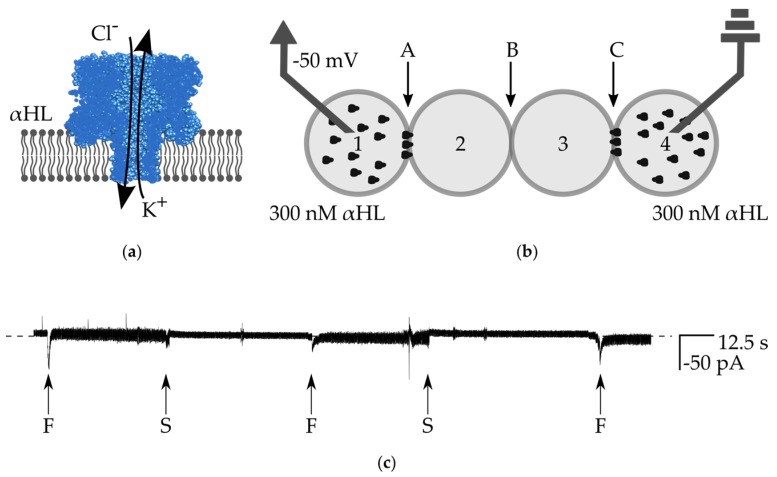
(**a**) Schematic drawing of α-hemolysin nanopores inserted in the lipid bilayer. The protein molecule assembles into a heptameric structure from water-soluble monomers. The channel non-selectively passes small molecules, including some ions; (**b**) schematic drawing of the experimental setup for testing the stability of the network. Droplets Nos. 1 and 4 contain 300 nM αHL, droplets Nos. 2 and 3 are composed of pure buffer. Bilayers are marked as A, B, and C; (**c**) ionic current recording from the voltage clamp experiment (−50 mV). The dashed line is a base level of current (0 pA). During the formation of bilayers (indicated with ”F”), we observe a short decrease of current, followed by a slightly higher current level, which results from higher electrical noise. In the experiment, we first apply the oil from the channel originating from inlet 6 and separate droplet No. 1 from droplet No. 2 (the rest of the network remains intact). The moment of disruption of bilayer A is marked with S (first from the left). After we stop the flow of oil, the network is reformed to its initial structure (middle “F”). Next, the second separation event follows—we separate droplet No. 4 from No. 3 and disrupt bilayer C (second “S” from the left). Next, the whole network is fully restored and the signal remains stable at the level of 0, indicating that there is no ionic conductance through the bilayer B. Moreover, by selective de-attachment of the outer droplets and subsequent reformation of the network, we prove that α-hemolysin nanopores do not escape from the original droplet.

We aimed to obtain an assembly of droplets in which the transmission of an electrical signal would be ensured within the whole network. The main goal was to measure the signal from the bilayer located in the center of the network—at the interface of droplets Nos. 2 and 3. In order to achieve this goal, the outer bilayers in the system need to exhibit high electrical conductivity. A properly formed lipid bilayer is impermeable to ions, however some peptides or proteins are able to spontaneously incorporate into the membrane and assemble pores enabling the transport of ions. An example of such protein is α-hemolysin (αHL)—an asymmetric, heptameric nanopore, which non-selectively passes small molecules, including potassium ions (Figure 4a).

We first determined the highest concentration of α-hemolysin nanopores, which does not lead to the disruption of the bilayer and coalescence of droplets. In order to do this, we performed measurements using the 2-droplet system, described previously [14]. We found that 9.9 µg/mL (300 nM) is the suitable concentration for the formation of stable bilayers comprising a high number of protein molecules in the bilayer for a sufficiently large current of ions (resistance is lower than 5 MΩ after several seconds), see Figure S3). However, in some experiments, especially after several exchanges of droplets, the pool of active channels in the droplets Nos. 1 and 4 can be depleted, and the edge bilayers can have higher resistance—e.g., the exemplary trace presented in Figure S4 indicates that the resistance of outer bilayers was around 20 MΩ.

In order to confirm the long-term stability of the network comprising highly perforated bilayers, we built an assembly in which droplets Nos. 1 and 4 contained a high concentration of αHL in buffer (300 nM αHL, 1 M KCl, 10 mM Hepes, pH 7), whereas droplets 2 and 3 are composed of buffer without protein (Figure 4b). We selectively de-attached droplets by applying the flow of oil from the thin perpendicular channels (flow of oil from inlet 6 resulted in the detachment of droplet No. 1 from the network, and flow from inlet 8 resulted in the de-attached of droplet No. 4). After switching off the flow of oil, we observed the reformation of the bilayers. In this experiment, we clamped the voltage at the constant level (−50 mV), however the formation of the bilayer is still visible as an increase of the level of noise in the recording of the electric current between the electrodes. In spite of the presence of potassium ions in all of the droplets, we did not observe any events attributed to the leakage of ions through the inner bilayer between droplets Nos. 2 and 3 (Figure 4c). It means that the bilayer B remains intact (it possesses an infinite resistance) and there is no sign of unwanted transfer of α-hemolysin nanopores from droplets Nos. 1 and 4 to droplets Nos. 2 and 3. It is worth noting that the transfer of αHL across the DIB is rather improbable due to the asymmetric structure of α-hemolysin nanopores (cap and barrel domains) [28].

### 3.3. Transmission of Signal through the Network

**Figure 5:**
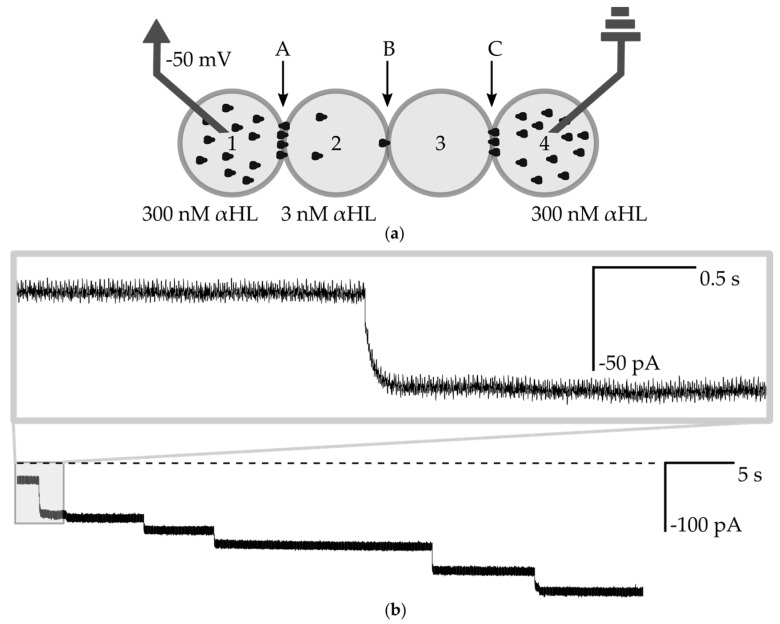
(**a**) Schematic drawing of the experimental setup for measuring the transmission of the signal through the network. Droplets Nos. 1 and 4 contain 300 nM αHL, droplet No. 2 contains 3 nM αHL and No. 3 is composed of pure buffer. Bilayers are marked as A, B, C; (**b**) Ionic current recording from the voltage clamp experiment (−50 mV). The dashed line is a base level of current (0 pA). The fragment shows step-changes of current, which indicate the insertion of αHL nanopores into the bilayer B. The incorporation of channels does not always contribute to 50 pA changes in the current, which is attributed to the variation between the structure or non-optimal assembly of individual pores [19]. Using the pre-assembled αHL heptamers instead of commercially available lyophilized protein should result in more uniform values of changes of current after the insertion of subsequent nanopores [14]. The inset depicts a single-channel insertion—the exponential shape of the signal is clearly visible.

In the next step, we intended to show the electrical communication within the network. Similar to the first experiment, we first introduced the outer (in the positions 1 and 4) droplets with the high concentration of αHL and the droplet with pure buffer is locked in position No. 3. Next, instead of the second inner droplet with pure buffer, we prepared a droplet containing a low concentration (0.01 µg/mL, 3 nM) of α-hemolysin and located this droplet in the network in position No. 2 (Figure 5a). Bilayers A and C possess relatively low electrical resistance due to their high number of pores that allow for a largely unrestricted flow of potassium ions. In the case of measurements in the classical 2-compartment system, the incorporation of a single α-hemolysin nanopore into a lipid bilayer results in a stepwise, square-shape increase of the current (−50 pA when −50 mV applied in 1 M KCl) [3]. In the network built from a higher number of lipid membranes, the voltage across the system is not constant—it is gradually redistributed among the bilayers [26]. Therefore, the incorporation events of a single αHL pore are recorded as exponential increases of the ionic current that do not immediately achieve a steady-state value (Figure 5b). The duration of the exponential decay phase depends on the resistance of two edge bilayers (A and C). We performed an analysis of the transient states of the system i.e., we derived the formula describing the time-dependent change of an ionic current upon insertion of an additional channel in the middle bilayer. Our formula requires a non-zero current at the time zero, so we assumed that there is already at least one nanopore present in the bilayer B i.e., *I*(*t* = 0) ≈ −50 pA. Our measurements are consistent with our calculations and the general performance of the system is similar to the one presented by Hwang et al. [25], (see Supplementary Materials for a model of the electric circuit, Figure S5). However, the edge bilayers (A and C) in our network have a lower resistance (more nanopores inserted) and consequently decay times are shorter than in the system presented by Hwang et al. We were able to exchange the droplet containing the low concentration of hemolysin and introduce fresh ones over a period of at least 1 h. We did not observe any continuous drop in current which would indicate the loss of activity of highly concentrated αHL trapped in outer droplets.

### 3.4. Measurements of the Interaction of a Nanopore with Small Molecules

Our system is also capable of testing the activity of inhibitors without direct contact of electrodes with the inner compartments of the network. So far, this feature has not been available in DIB microfluidic systems. In combination with the on-demand exchange of droplets in the network, the measurement of inhibitors activity without the need for electrode contact has potential for performing long-term screening of interactions of small molecules with nanopores without the risk of adsorption of the tested chemicals on the electrodes and unwanted carryover of compounds between the tested droplets. As a consequence, there is no need to cyclically wash electrodes with pure buffer which was necessary in the 2-droplet system, presented previously [14].

**Figure 6:**
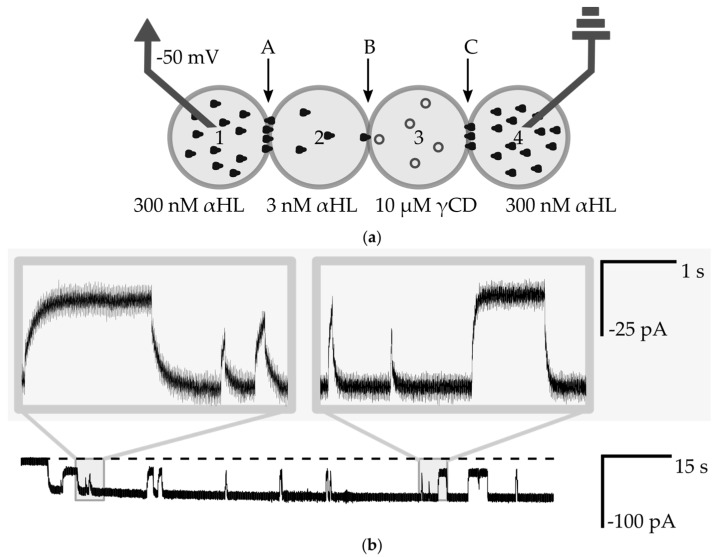
(**a**) Schematic drawing of the experimental setup for measurements of the interaction of a nanopore with small molecules. Droplets Nos. 1 and 4 contain 300 nM αHL, droplet No. 2 contains 3 nM αHL and No. 3 contains 10 µM γ-CD. Bilayers are marked as A, B, C; (**b**) Ionic current recording from voltage clamp experiment (−50 mV). The dashed line is a base level of current (0 pA). The selected fragment shows interactions between a single αHL channel with γCD molecules. The level of app. −50 pA is attributed to the open-channel conformation, whereas transient changes to the level of −20 pA come from the steric inhibition of the flow of ions by γCD molecules. The insets show the exponential nature of changes in the level of ionic current.

In order to confirm the capability of screening of inhibitors, we formed a droplet containing 10 µM γ-cyclodextrin (γCD) and locked this droplet in the network in position No. 3 (Figure 6a). γ-cyclodextrin is a cyclic sugar and a non-covalent reversible blocker of α-hemolysin. The binding of the inhibitor inside of αHL nanopore causes transient decreases of the current by about 60% of the open pore value [3]. The current trace from interactions of a single αHL channel (present in bilayer B) with γCD molecules is depicted in Figure 6b. Very short events of pore inhibition did not always reach a value of 60% of the current. A system requires time to establish a new steady-state, and detection of short changes is limited by decay phases that depend on the resistance of edge bilayers. However, from the analysis presented in Supplementary Materials (Figures S4 and S5), we can safely assume that the decay time is shorter that 0.1 s and it is much shorter than in previous analyses of membrane protein inhibition in the networks e.g., the system presented by Hwang et al. Nanopores in the bilayer C are also transiently inhibited, however the high number of channels in the edge bilayer act as resistors connected in parallel. Each of the channels present in the edge bilayer contributes to less than 0.25% of the transmission of the total current. The inhibition of one of the channels in bilayer C results in the drop of measured current by only 0.15 pA. Taking into account the level of electric noise, such small changes are not noticeable in the measured signal.

A full screening of kinetics requires that many concentrations of an inhibitor are tested. Our system can be easily interfaced with an external generation of dilutions either by a robot [14,29] or in a separate microfluidic device or module [25,30]. Moreover, selected droplets can be removed from the network in an intact form and they can be directed for further experimentation. This could find an application in the more detailed studies of transport across the lipid bilayer [31,32]. In this demonstration, we rely on α-hemolysin nanopores as a means to provide high permeability of bilayers. We did not observe a significant depletion of monomers even after long-term operation of the device (see Figure S3), however the insertion rate slightly decreased in subsequent bilayers formed between the sample droplets and the same droplet with a high concentration of nanopores. Other types of molecules, such as artificial nanopores [33,34], might also be used to ensure the communication within the network. The screening capabilities are limited by the time needed for the formation of the bilayer (60 s) and the duration of the actual measurement, since the removal of a droplet from a network and replacement with a new one took less than 20 s. The throughput of the system can be further increased through the parallelization of microfluidic automation [35] and by simultaneous multiple electrophysiological measurements [13,22].

## 4. Conclusions

We have developed a novel microfluidic chip dedicated to the formation of networks built from droplets interconnected by lipid bilayers. The automated operation of the device with external electromagnetic valves allows for a precise control of the flow of fluids on the chip. Most importantly, the droplets within the network can by individually addressed—generated on a chip, transported to the hydrodynamic trap for the formation of the bilayer, incubated and removed from the network without disrupting the rest of the structure. We demonstrated that membrane proteins, in that case α-hemolysin nanopores, may be used to functionalize bilayers in the network, so that the electrical communication within the network is achieved. The activity of single channels can be measured within the network formed of four droplets containing various concentrations of nanopores in such a way that the risk of cross contamination between successive measurements is eliminated.

## Supplementary Materials

The following are available online at www.mdpi.com/2072-666X/8/3/93/s1; Figure S1: Design and operation of microfluidic device. Figure S2: Regulation of the size of bilayers and on demand separation of droplets. Figure S3: Measurements of the dynamics of incorporation of high concentration of α-hemolysin into bilayers. Figure S4: The theoretical changes of ionic current upon insertion of second αHL nanopore to the middle bilayer for various resistances of each of edge bilayers. Figure S5: Model of electric circuit built from 4 droplets Video S1: Formation of the network in a trap—exchange of one of the droplets within the network. Scale bar is 500 μm, and video is speeded up 4×. Video S2: Selective de-attachment of droplets within the network, scale bar is 500 μm and the video is in real-time.
