# Supplementary material for: An Automated Microfluidic System for the Generation of Droplet Interface Bilayer Networks
Source: Micromachines (Basel). 2017 Mar 21;8(3):93. doi: 10.3390/mi8030093 (PMC6190347; doi:10.3390/mi8030093)
Supplement: Supplementary file 1 [file micromachines-08-00093-s001.zip › micromachines-178560 suppl final.pdf]

Supplementary Materials: An Automated Microfluidic System for the Generation of Droplet Interface Bilayer Networks

Magdalena A. Czekalska, Tomasz S. Kaminski, Michal Horka, Slawomir Jakiela and Piotr Garstecki

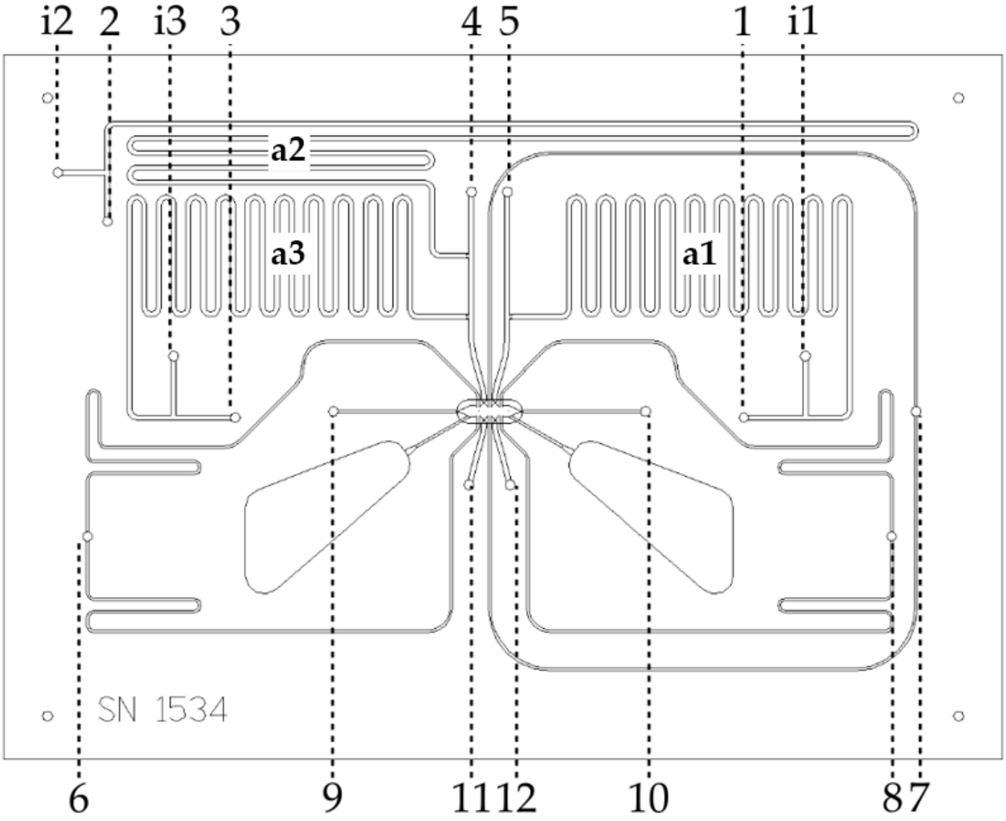

Figure S1. Design and operation of microfluidic device. Detailed diagram of the layout of the microfluidic chip. The numbers indicate: "1–8"—inlets for oil, "9–12"—outlets, "a1–a3" —aspiration modules for aqueous samples, "i"—inlets for aqueous samples, "i1"—buffer/solution of inhibitor, "i2"—solution with high concentration of α-hemolysin (300 nM), "i3"—solution with low concentration of α-hemolysin (3 nM). The dimensions of a whole chip are 82 mm × 53 mm.

Operation of the device: Before performing the experiments, we first filled the whole chip with a continuous organic phase containing lipids (1 mg·mL^{-1} of 1,2-diphytanoyl-snglycero-3-phosphocholine (DPhPC) dissolved in 75% hexadecane and 25% silicone oil (AR20). Twelve valves (V165, equipped with Z070D coils, Sirai, Bussero, Italy) were controlled with a custom written Lab View script via a National Instruments card (NI PCIE-6321, Austin, TE, USA). The value of pressure (500 mbar) applied to the oil reservoirs was set using manual pressure regulators (PR1-RGP, Bosch Rexroth, Lohr am Main, Germany) and monitored using digital manometers (AZ 82100, AZ Instruments, Taichung, Taiwan). Flow of oil from inlets 6–8 was controlled by a separate pressure controller and lower pressure was applied in that case (150 mbar). The oil was delivered from pressurized reservoirs, through the valves (numbered 1–8), next through the steel capillaries and onto the chip. Four outlets were connected via PTFE tubing to valves 9–12 in order to control the flow through the outlets.

The aqueous samples (~50 µL of protein solution, inhibitor solution or buffer) were deposited into long storage channels on the chip from glass syringes (1750SL Gastight, Hamilton, Reno, NE, USA) that were next closed with build-in valves after the deposition of the sample. Once the liquids were introduced on the chip, the flow of oil was initiated to push the face of sample plugs towards the T-junctions. In order to generate a droplet, we applied the flow of oil from inlets (either 1, 2 or 3)

that pushed a defined volume of a sample (500 nL) into the orthogonal channel, then broken it off with the flow of oil from inlet 4 or 5 and transported the droplet into the trap. In the experiments presenting the transmission of the signal through the network, we first generated two droplets containing 300 nM α-hemolysin and locked them in the chambers at the edges of a trap. Then we generated droplets either containing buffer, 3 nM αHL or 10 μM γ-cyclodextrin.

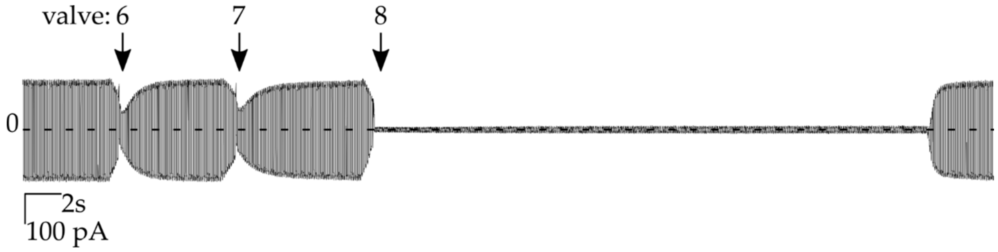

Figure S2. Regulation of the size of bilayers and on demand separation of droplets. An example of recording of the capacitive current, which is proportional to the size of a bilayer. We applied the flow of oil from respective valves (Nos. 6, 7 or 8, see Figure 1 for the scheme of chip). Oil flew through the thin channels, perpendicular to the long axis of the droplet network, and gently pushed droplets apart (Nos. 6 and 7). If the flow of oil is applied for enough long period of time, it detaches the droplets completely (8). See the Video S2 presenting the execution of the experiment.

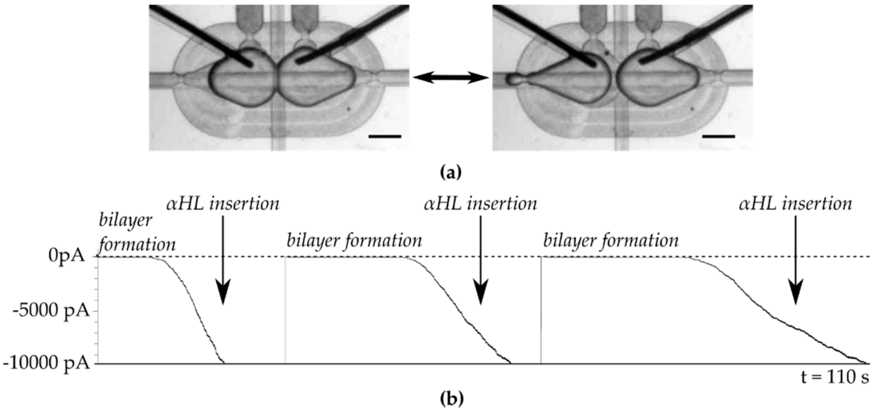

Figure S3. Measurements of the dynamics of incorporation of high concentration of α-hemolysin into bilayers. In this experiment we tested high concentration of αHL (300 nM) using the 2-droplet system. The aim of this measurement was to verify whether: (i) the lipid bilayer withstands the insertion of high number of channels, (ii) the droplets can be separated without the coalescence, (iii) after the reformation of bilayer there is enough number of pores available for insertion into the membrane. (**a**) Micrographs of experimental setup. The bilayer was formed at the interface of two droplets, each composed of 1 M KCl, 10 mM Hepes. One of the droplets contained 300 nM αHL. First, the droplets were placed in the trap, and next we performed several times a 3-step procedure: (i) wait for the formation of a bilayer, (ii) measure the electrical signal and (iii) separate the droplets by applying the flow of oil from the thin channel visible in the center of the photographs. Scale bar is 500 μm. (**b**) Current recording from the voltage clamp (−50 mV) experimental procedures described in (a). After the formation of a bilayer, the current decreased gradually due to the insertion of subsequent nanopores. The maximum current that can be recorder by Axopatch instrument is 10 nA, which indicates that more than 200 pores were present in the bilayer (1 pore gives the change of current equal −50 pA). After the signal reaches the maximum capacity of the Axopatch, we separated the droplets and allowed for reformation of the bilayer. The measurement took place after 1.5 h incubation of a sample on the chip, which indicates that solution with high concentration of αHL monomers has long-term capability for efficient insertion of pores to the membrane.

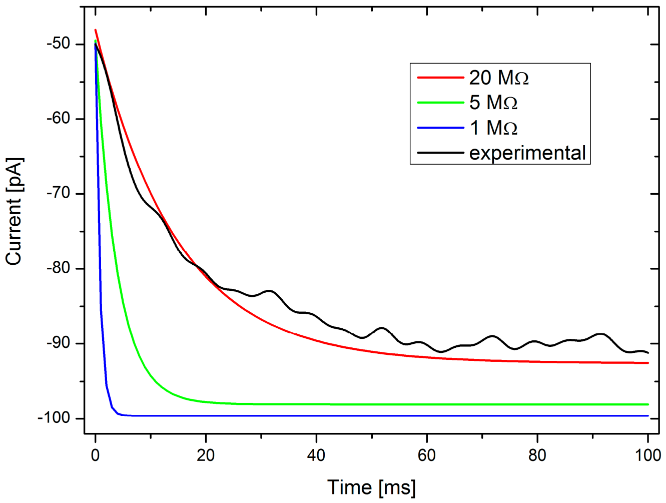

Figure S4. The theoretical changes of ionic current upon insertion of second αHL nanopore to the middle bilayer for various resistances of each of edge bilayers. The resistances of 20, 5 and 1 MΩ correspond to respectively ca. 50, 200 and 1000 αHL inserted to each of two edge bilayer (A and C). The black line represents exemplary current recordings of the insertion of protein nanopore. In this case the experimental trace is similar to the theoretical curve for the resistance of outer bilayer that equals 20 MΩ, thus indicating that there were around 50 nanopores already inserted in each of the outer bilayers

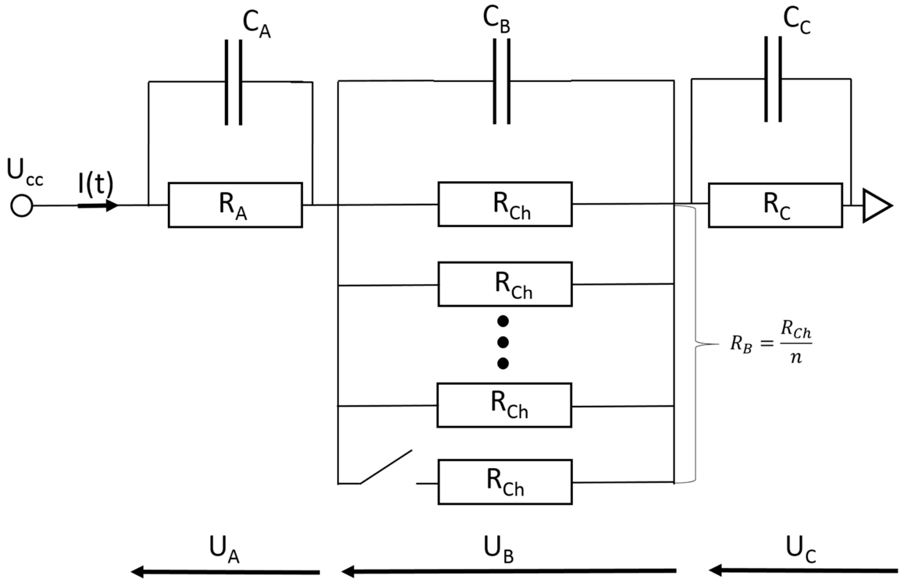

Figure S5. Model of electric circuit built from 4 droplets. R_A, R_B, R_C—total electrical resistances of the bilayers A, B and C, respectively; R_{Ch}—resistance of the single αHL nanopore; U_A, U_B, U_C—voltage drops across the subsequent bilayers: A, B and C, respectively; n—number of nanopores inserted in the bilayer B; U_{CC}—Electrical potential applied to the droplet interface bilayer (DIB) network.

Assuming that $R_A \approx R_C \ll R_B$ and $C_A \approx C_B \approx C_C$ we could claim that any changes of voltages (drops U_A and U_C) due to the insertion of additional pores in the bilayer B may be described by $U_A(t) = R_A I(t)$ and $U_C(t) = R_C I(t)$. Therefore, we can simplify an electric circuit by omitting capacitors C_A and C_C. To calculate $I(t)$ we can write following formulas:

$$U_{CC} = U_A(t) + U_B(t) + U_C(t) = R_A I(t) + R_C I(t) + U_B(t) \quad (1)$$

Hence,

$$I(t) = \frac{U_{CC} - U_B(t)}{R_A + R_C} \tag{2}$$

Furthermore, we can also write

$$I(t) = C_B \frac{dU_B(t)}{dt} + \frac{U_B(n+1)}{R_{Ch}} \tag{3}$$

Combining the Equations (2) and (3) and calculating the differential equation we obtain following formula:

$$U_B(t) = \frac{\alpha - \sigma}{\beta} e^{-\beta t} \tag{4}$$

where

$$\alpha = \frac{U_{CC}}{(R_A+R_C)C_B}, \beta = \frac{1}{C_B}\left(\frac{1}{R_A+R_C} + \frac{n+1}{R_{Ch}}\right)$$

and

$$\sigma = \frac{U_{CC}}{(R_A+R_C)C_B} - \frac{1}{C_B}\left(\frac{1}{R_A+R_C} + \frac{n+1}{R_{Ch}}\right)\frac{U_{CC}}{R_A + R_B + \frac{R_{Ch}}{n}}\frac{R_{Ch}}{n}$$

According to the fact that $U_C \approx U_A$ we can write that

$$I(t) = \frac{0.5(U_{CC} - U_B(t))}{R_A} = \frac{1}{2R_A}\left(U_{CC} - \frac{\alpha}{\beta} + \frac{\sigma}{\beta} e^{-\beta t}\right) \tag{5}$$

From the Equation (5) we can see that the time decay is described by β, which is the function of C_B, R_A, R_C, R_{Ch} and n. As a consequence, we can assume that increasing resistances R_A and R_C results in the longer time needed for change of current after creation of new pore. With resistances going to zeros the current response is going to be stepwise what was and presented previously in several demonstrations of a simple double droplets network.

Video S1: Formation of the network in a trap—exchange of one of the droplets within the network. Scale bar is 500 µm. Video speeded up 4×.

Video S2: Selective de-attachment of droplets within the network. Scale bar is 500 µm and the video is in real-time.

© 2017 by the authors. Submitted for possible open access publication under the terms and conditions of the Creative Commons Attribution (CC BY) license (http://creativecommons.org/licenses/by/4.0/).
